# A pioneer survey and DNA barcoding of some commonly found gastropod molluscs on Robben Island

**DOI:** 10.3897/zookeys.481.8188

**Published:** 2015-02-04

**Authors:** Herman van der Bank, Richard Greenfield

**Affiliations:** 1 African Centre for DNA Barcoding, Department of Zoology, University of Johannesburg, APK Campus, PO Box 524, Auckland Park, 2006, South Africa

**Keywords:** Mollusca, Gastropoda, mitochondrial gene COI, species identification

## Abstract

Nineteen species of abundant gastropods were collected at Robben Island, including introduced dune snails and European brown garden snails. They were identified using morphology and DNA barcoding. It was expected that the species recorded would be similar to those from the Cape peninsula, South Africa, but we were surprised to find some exceptions: the very abundant invasive mussel species in South Africa, the South American bisexual mussel (*Semimytilus algosus*), and the beaded topshells (*Oxystele impervia*) were not found on Robben Island. Possible explanations are presented for these differences.

## Introduction

Robben Island is most renowned for its maximum security prison, which housed President Nelson Mandela of SA. Robben Island is 7 km from Bloubergstrand (SA), the length of Robben Island is 5.4 km, the width is 2.5 km, and has an area of 574 hectares (approximately 5.74 km^2^) with the highest point at 30 m above sea level and an annual rain fall of between 300 mm and 400 mm. It is less known for being the island to which lepers were banned to become miserable island outcasts. The leper colony was initiated in 1846 with special water police patrols to ensure no escapees. Robben Island has recently become a tourist attraction, possibly contributing towards the decline of African penguin numbers to the extent that they became an endangered species (Weller et al. 2014).

Unfortunately, little research on molluscs has been carried out on the island. The only available reference is to one mussel species at Robben Island by Pollock (1979). The aim of this study is to report results from a survey of the biodiversity, confirmed by voucher identifications and DNA barcoding of the common molluscs of Robben Island as part of the international Barcoding of Life Data Systems (BOLD; http://www.barcodinglife.org) project. We were very interested to determine the extent to which alien species have invaded the island, as alien invasive species are becoming a big problem in SA (Picker and Griffiths 2011). The South American bisexual mussel is especially worrisome because it proliferates extremely rapidly and is replacing endemic mussel species in South Africa.

## Materials and methods

Samples were collected from random sampling sites during July 2011. Standard DNA barcoding protocols were used to ensure correct identification of individuals (e.g. Van der Bank et al. 2013).

DNA extraction, polymerase chain reactions (PCR) and sequencing of the COI region (animal DNA barcode) were done at the Canadian Centre for DNA Barcoding (CCDB). Standard CCDB protocols for PCR reactions were followed as described by Hajibabaei et al. (2005). Multiple Sequence Comparison by Log-Expectation (MUSCLE vs. 3.8.31, Edgar 2004 was performed) for sequence alignment. GenBank accession numbers, BOLD process identification numbers and voucher information are all available online (www.boldsystems.org). The Kimura 2-parameter (K2P) model (Kimura 1980) was used to measure genetic distances.

We reconstructed Bayesian phylogenetic trees using MrBayes v3.1.2 (Ronquist and Huelsenbeck 2003). jModelTest v0.1.1 (Posada 2008) under the Akaike information criterion (Posada and Buckley 2004) was used as the best-fit model of DNA sequence evolution. The Bayesian tree was generated by selecting the TrN + I model and nine million generations, with sampling one tree every 100 generations, was used in the analysis.

PAUP* v4.10b10 (Swofford 2002) was used for maximum parsimony (MP) analyses and the data and tree searches were done using heuristic searches with 1 000 random sequence additions keeping only 10 trees. All character transformations were treated as equally likely i.e. Fitch parsimony (Fitch 1971) for the tree bisection-reconnection as performed with MP searches and bootstrap resampling (Felsenstein 1985) done using PAUP* v4.10b10 (Swofford 2002). The American bisexual mussel (*Semimytilus algosus* (Gould, 1850); BOLD process Id: HvdB-12-2010-116) was used as outgroup.

**Figure 1.**
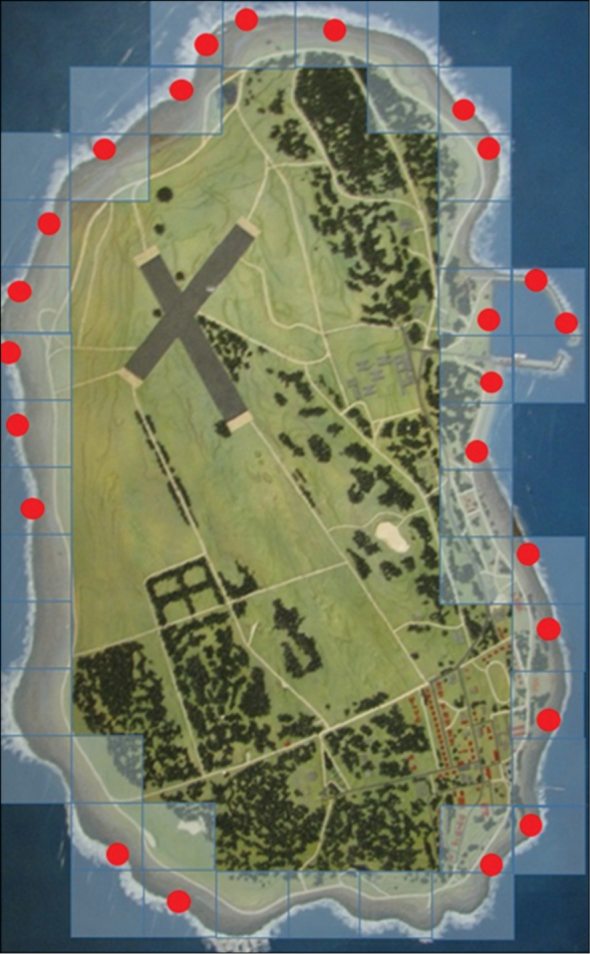
Random sampling sites on Robben Island from which species were collected.

Depending on availability, up to 10 individuals per species were collected (Table 1) from random sampling sites (Figure 1) generated with the aid of computer software (http://www.random.org/). Some terrestrial, but mostly marine snails were collected from the *Littorina* to *cochlear* zones. Data capture as prescribed by BOLD (including GPS, altitude, temperature, photographs of the localities and species, and voucher information) was done. Voucher specimens (shells) were also collected and deposited at the KwaZulu-Natal Museum (SA) in an attempt to limit future bio-prospecting.

**Table 1.** BOLD process and sample identity numbers, species collected, authorities and families. **Bold font** indicates samples for which no DNA barcodes were obtained from BOLD.

BOLD Process	Sample identity	Species	Family
HVDBM476-11 HVDBM495-11	HVDBM-ROB-96 HVDBM-ROB-97	*Afrolittorina knysnaensis* (Philippi, 1847)	Littorinidae
HVDBM569-11	**HVDBM-ROB-94**	*Burnupena catarrhacta* (Gmelin, 1791)	Buccinidae
HVDBM528-11 HVDBM529-11 HVDBM530-11 HVDBM531-11 HVDBM532-11	HVDBM-ROB-53 HVDBM-ROB-54 HVDBM-ROB-55 HVDBM-ROB-56 HVDBM-ROB-57	*Burnupena cincta* (Röding, 1798)	Buccinidae
HVDBM533-11 HVDBM534-11 HVDBM535-11 HVDBM536-11 HVDBM537-11	HVDBM-ROB-58 HVDBM-ROB-59 HVDBM-ROB-60 HVDBM-ROB-61 HVDBM-ROB-62	*Cornu aspersum* (Müller, 1774)	Helicidae
HVDBM525-11 HVDBM526-11 HVDBM527-11 HVDBM553-11 HVDBM554-11 HVDBM555-11 HVDBM556-11 HVDBM568-11	HVDBM-ROB-50 HVDBM-ROB-51 **HVDBM-ROB-52** HVDBM-ROB-78 HVDBM-ROB-79 HVDBM-ROB-80 HVDBM-ROB-81 HVDBM-ROB-93	*Crepidula complanata* (Krauss, 1848)	Calyptraeidae
HVDBM496-11 HVDBM497-11 HVDBM552-11	HVDBM-ROB-21 HVDBM-ROB-22 HVDBM-ROB-77	*Cymbula compressa* (Linnaeus, 1758)	Patellidae
HVDBM481-11 HVDBM482-11 HVDBM483-11 HVDBM484-11 HVDBM485-11 HVDBM486-11 HVDBM500-11 HVDBM540-11 HVDBM541-11 HVDBM542-11	**HVDBM-ROB-06** HVDBM-ROB-07 HVDBM-ROB-08 HVDBM-ROB-09 HVDBM-ROB-10 HVDBM-ROB-11 HVDBM-ROB-25 HVDBM-ROB-65 HVDBM-ROB-66 HVDBM-ROB-67	*Cymbula granatina* (Linnaeus, 1758)	Patellidae
HVDBM487-11 HVDBM488-11 HVDBM489-11 HVDBM539-11	HVDBM-ROB-12 HVDBM-ROB-13 HVDBM-ROB-14 HVDBM-ROB-64	*Cymbula oculus* (Born, 1778)	Patellidae
HVDBM499-11 HVDBM570-11	HVDBM-ROB-24 HVDBM-ROB-95	*Fissurella mutabilis* (Sowerby, 1835)	Fissurellidae
HVDBM561-11	HVDBM-ROB-86	*Helcion pectunculus* (Gmelin, 1791)	Patellidae
HVDBM506-11 HVDBM507-11 HVDBM508-11 HVDBM509-11 HVDBM510-11	HVDBM-ROB-31 HVDBM-ROB-32 HVDBM-ROB-33 HVDBM-ROB-34 HVDBM-ROB-35	*Oxystele tigrina* (Anton, 1839)	Trochidae
HVDBM511-11 HVDBM512-11 HVDBM513-11 HVDBM514-11 HVDBM515-11	HVDBM-ROB-36 HVDBM-ROB-37 HVDBM-ROB-38 HVDBM-ROB-39 HVDBM-ROB-40	*Oxystele variegata* (Anton, 1839)	Trochidae
HVDBM498-11	HVDBM-ROB-23	*Scutellastra barbara* (Linnaeus, 1758)	Patellidae
HVDBM477-11 HVDBM478-11 HVDBM479-11 HVDBM480-11	**HVDBM-ROB-02** **HVDBM-ROB-03** **HVDBM-ROB-04** **HVDBM-ROB-05**	*Scutellastra cochlear* (Born, 1778)	Patellidae
HVDBM490-11 HVDBM491-11 HVDBM492-11 HVDBM493-11 HVDBM494-11 HVDBM562-11	HVDBM-ROB-15 HVDBM-ROB-16 HVDBM-ROB-17 HVDBM-ROB-18 HVDBM-ROB-19 **HVDBM-ROB-87**	*Scutellastra granularis* (Linnaeus, 1758)	Patellidae
HVDBM563-11 HVDBM564-11 HVDBM565-11 HVDBM566-11	HVDBM-ROB-88 HVDBM-ROB-89 **HVDBM-ROB-90** HVDBM-ROB-91	*Siphonaria serrata* (Fischer von Waldheim, 1807)	Siphonariidae
HVDBM501-11 HVDBM502-11 HVDBM503-11 HVDBM504-11 HVDBM505-11	HVDBM-ROB-26 HVDBM-ROB-27 HVDBM-ROB-28 HVDBM-ROB-29 HVDBM-ROB-30	*Siphonaria oculus* (Krauss, 1848)	Siphonariidae
HVDBM538-11 HVDBM1129-12 HVDBM1130-12 HVDBM1131-12 HVDBM1132-12 HVDBM1133-12	HVDBM-ROB-63 HVDBM_ROB_1010 HVDBM_ROB_1011 HVDBM_ROB_1012 HVDBM_ROB_1013 HVDBM_ROB_1014	*Theba pisana* (Müller, 1774)	Helicidae
HVDBM1134-12 HVDBM1135-12 HVDBM1136-12 HVDBM1137-12 HVDBM1138-12	HVDBM_ROB_1015 HVDBM_ROB_1016 HVDBM_ROB_1017 HVDBM_ROB_1018 **HVDBM_ROB_1019**	*Trigonephrus globulus* (Müller, 1774)	Dorcasiidae
HVDBM551-11	HVDBM-ROB-76	*Turbo cidaris* (Gmelin, 1791)	Turbinidae

An unedited BOLD identification tree of barcoded southern African individuals is available from the corresponding author. This includes 815 sequenced individuals from 184 species, 118 genera, and 76 families in southern Africa (excluding the ones that were published already in other journals such as in Van der Bank et al. 2013).

## Results and discussion

BOLD process and sample identity numbers, species collected, authorities and families are listed in Table 1. We did not receive DNA barcodes from BOLD for Burnupena catarrhacta (Röding, 1798) and Scutellastra cochlear (Born, 1778), and for a few individuals from other species (sample identity numbers in bold, Table 1).

**Figure 2.**
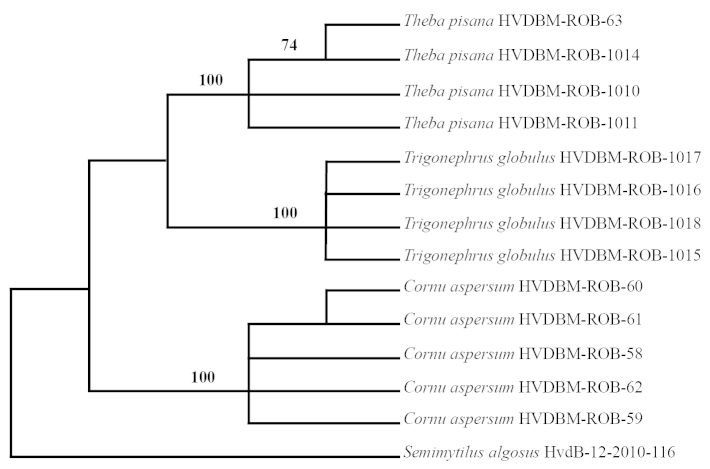
A parsimonious bootstrap (50% majority-rule) consensus tree obtained for the land snails, using *Semimytilus algosus* as the outgroup.

**Figure 3.**
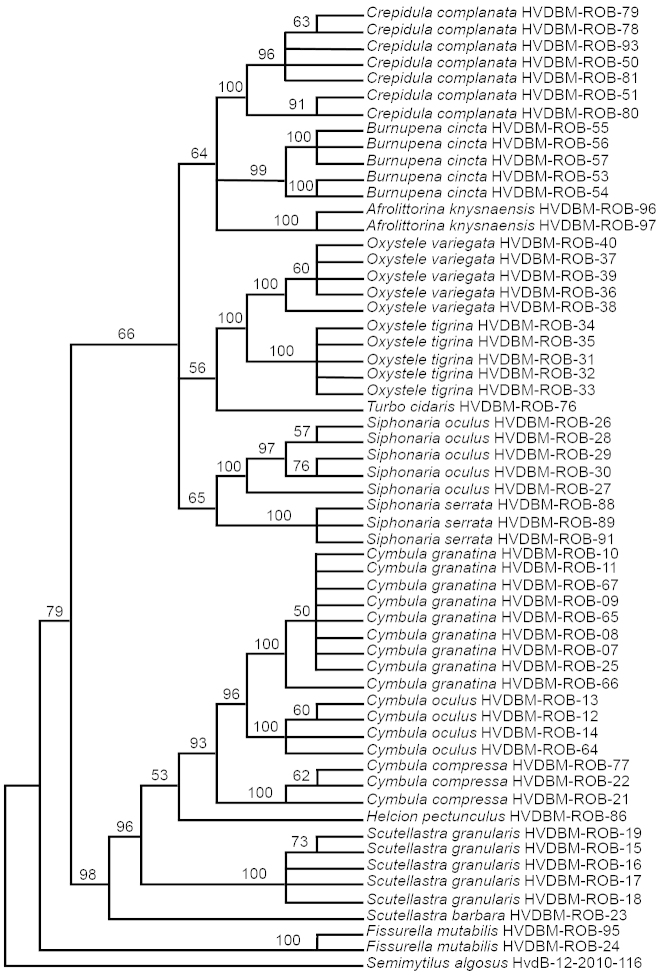
A parsimonious bootstrap (50% majority-rule) consensus tree obtained for the marine snails, using *Semimytilus algosus* as the outgroup.

The most abundant terrestrial snail we encountered was *Theba pisana* (Müller, 1774), an introduced dune snail. As expected, more Cape endemic terrestrial snails were found (*Trigonephrus globulus*, Müller, 1774) and fewer introduced European brown garden snails (*Cornu aspersum*, Müller, 1774, formerly *Helix aspersa*) were recorded. Figure 2 indicates that the land snail species are monophyletic and were well-supported (bootstrap values 74-100%; average 93.5% using the South American bisexual mussel as outgroup, also in Figure 3 for the marine molluscs). The aligned COI matrix is 654 base pairs long, the numbers of constant characters are 335; 319 characters are variable (of which 146 are parsimony uninformative and 173 are parsimony informative). The tree length is 414 steps with a consistency index of 0.942 and a retention index of 0.963. Mean intra-specific divergence is 0.15 and 24.5 for the mean distance to nearest neighbour. This indicates that there is a barcode gap (Meyer and Paulay 2005) in the dataset, thus confirming COI as an appropriate DNA region for taxon identification within these studied molluscs.

Approximately 85–90% of the habitat at the coast consists of rocky shores, with low biodiversity of sparsely populated barnacles and molluscs (bivalves, limpets, and mussels): it consisted mostly of individuals of the same species. According to the island conservationists, this can be attributed to the rough seas.

We were pleasantly surprised not to have found any South American bisexual mussel on Robben Island, as they are extremely common on the SA coastline (only approximately 6.9 km away) and Robben Island was joined with SA approximately 10 000 years ago (Thackeray 2001). They are extremely prolific breeders and are a threat to SA and Namibian mussels because they outcompete endemic mussel species. They only grow to 5 cm (Branch et al. 2010) and are therefore not suitable as a human food source as are the endemic species. It is conceivable that the Benguela Upwelling System might be responsible for their offspring to be transported away from the island in a more northerly direction. The aligned COI matrix in Figure 3 is 654 base pairs long, the numbers of constant characters are 258, and 396 characters are variable (of which 51 are parsimony uninformative and 345 are parsimony informative). The tree length is 1599 steps with a consistency index of 0.439 and a retention index of 0.862. Mean intra-specific divergence is 0.79 and 20.04 for the mean distance to nearest neighbour. This, again, indicates that there is a barcode gap in the dataset; thus confirming COI as an appropriate DNA region for taxon identification for the marine molluscs.

At the generic level, all of the marine species are monophyletic and were well supported (bootstrap values 98–100%; Figure 3), except for the single representatives of *Helcion pectunculus* (Gmelin, 1791) and *Turbo cidaris* (Gmelin, 1791) (bootstrap values 53% and 56% respectively). *Helcion* clusters as a sister group to Cymbula, as also reported by Nakano and Ozawa (2007).

We were also surprised not to find any *Oxystele impervia* (Menke, 1843) individuals on the island, which could be due to differences in habitat preferences: they are found more abundantly higher up the shore than, for example, *Oxystele variegata* (Heller and Dempster 1991), and possibly avoided the lower shore when SA and Robben Island were linked. It is also possible that the Benguela Upwelling System might prevent the free-swimming larvae of *Oxystele impervia* to reach the island, but it is more likely that thorough sampling is required before this can be confirmed.

The slipper-limpet (*Crepidula complanata* Krauss, 1848) was found with smaller males on top of the larger females; both live on other shells but are not parasites. Two clades with 91% and 97% bootstrap support were obtained (Figure 3). One specimen of *Cymbula granatina* (Linnaeus, 1758; HVDBM-ROB-66) was found that differed only by one sequence from the other *Cymbula granatina* individuals.

Almost no molluscs occurred on the man-made structures surrounding the harbour, most probably because these were installed recently, in 2004. This area was studied because introductions from ships (which transport tourists from SA as well as island staff and children) are most likely to occur at the harbour.

Surveys that are much more detailed are required to report on the biodiversity of the island; however, this is the first study to link DNA sequence results with the morphology of the commonly found molluscs on Robben Island and thereby to contribute to the global biodiversity fauna data that could enhance future efforts in conservation and management.

## References

[B1] Branch GM Griffiths CL Branch ML Beckley LE (2010) Two Oceans – A Guide to the Marine Life of Southern Africa. Random House Struik (Pty) Ltd., Cape Town, South Africa, 456 pp.

[B2] Edgar RC (2004) MUSCLE: Multiple sequence alignment with high accuracy and high throughput. Nucleic Acids Research 32: 1792–1797. doi: 10.1093/nar/gkh340 15034147 10.1093/nar/gkh340 PMC390337

[B3] Felsenstein J (1985) Confidence levels on phylogenies: an approach using the bootstrap. Evolution 39: 783–791. doi: 10.2307/2408678 10.1111/j.1558-5646.1985.tb00420.x 28561359

[B4] Fitch WM (1971) Towards defining the course of evolution: minimum change for a specific tree topology. Systematic Zoology 20: 406–416. doi: 10.2307/2412116

[B5] Hajibabaei M De Waard JR Ivanova NV Ratnasingham S Dooh RT Kirk SL Mackie PM Hebert PDN (2005) Critical factors for assembling a high volume of DNA barcodes. Philosophical Transactions of the Royal Society B 360: 1959–1967. doi: 10.1098/rstb.2005.1727 10.1098/rstb.2005.1727 PMC1609220 16214753

[B6] Heller J Dempster Y (1991) Detection of two coexisting species of *Oxystele* (Gastropoda, Trochidae) by morphological and electrophoretic analysis. Journal of Zoology 223: 395–418. doi: 10.1111/j.1469-7998.1991.tb04773.x

[B7] Kimura M (1980) A simple method for estimating evolutionary rates of base substitutions through comparative studies of nucleotide sequences. Journal of Molecular Evolution 16: 111–120. doi: 10.1007/BF01731581 7463489 10.1007/BF01731581

[B8] Meyer CP Paulay G (2005) DNA barcoding: error rates based on comprehensive sampling. PLoS Biology 3: 2229–2238. doi: 10.1371/journal.pbio.0030422 10.1371/journal.pbio.0030422 PMC1287506 16336051

[B9] Nakano T Ozawa T (2007) Worldwide phylogeography of limpets of the order Patellogastropoda: molecular, morphological and palaeontological evidence. Journal of Molluscan Studies 73: 79–99. doi: 10.1093/mollus/eym001

[B10] Picker M Griffiths CL (2011) Alien and Invasive Animals – A South African Perspective. Random House Struik (Pty) Ltd., Cape Town, South Africa, 240 pp.

[B11] Pollock DE (1979) Predator-prey relationships between the rock lobster *Jasus lalandii* and the mussel *Aulacomya ater* at Robben Island on the Cape west coast of Africa. Marine Biology 52: 347–356. doi: 10.1007/BF00389076

[B12] Posada D (2008) jModelTest: phylogenetic model averaging. Molecular Biology and Evolution 25: 1253–1256. doi: 10.1093/molbev/msn083 18397919 10.1093/molbev/msn083

[B13] Posada D Buckley TR (2004) Model selection and model averaging in phylogenetics: advantages of Akaike information criterion and Bayesian approaches over likelihood ratio tests. Systematic Biology 53: 793–808. doi: 10.1080/10635150490522304 15545256 10.1080/10635150490522304

[B14] Ronquist F Huelsenbeck JP (2003) MrBayes 3.1.2: Bayesian phylogenetic inference under mixed models. Bioinformatics 19: 1572–1574. doi: 10.1093/bioinformatics/btg180 12912839 10.1093/bioinformatics/btg180

[B15] Swofford DL (2002) PAUP*: phylogenetic analysis using parsimony (* and other methods), version 4.10. Sinauer, Sunderland, Massachusetts.

[B16] Thackeray F (2001) Robben Island and past climatic changes. http://www.neuronet.co.za/robben.html [accessed 2/June 2014]

[B17] Van der Bank FH Herbert D Greenfield R Yessoufou K (2013) Revisiting species delimitation within the genus *Oxystele* using DNA barcoding approach. ZooKeys 365: 337–354. doi: 10.3897/zookeys.365.5356 24453566 10.3897/zookeys.365.5356 PMC3890686

[B18] Weller F Cecchini L Shannon L Sherley RB Crawford RJM Altwegg R Scott L Stewart T Jarre A (2014) A system dynamics approach to modelling multiple drivers of the African penguin population on Robben Island, South Africa. Ecological Modelling 277: 38–56. doi: 10.1016/j.ecolmodel.2014.01.013

